# Supplementary material for: UK biobank: Enhanced assessment of the epidemiology and long-term impact of coronavirus disease-2019
Source: Camb Prism Precis Med. 2023 Aug 29;1:e30. doi: 10.1017/pcm.2023.18 (PMC10953745; doi:10.1017/pcm.2023.18)
Supplement: Supplementary file 1 [file S2752614323000182sup001.pdf]

UK Biobank: enhanced assessment of the epidemiology and long-term impact of COVID-19

Supplementary Appendix, Table of contents

	Page
**Table S1: Baseline and resurvey assessment in UK biobank**	2
**Table S2: Health record linkage in UK biobank**	3
**Table S3: List of the COVID-19 related publication that used UK Biobank data resources.**	4
**Figure S1: UK Biobank SARS-CoV-2 Serology Study**	20
**Figure S2: UK Biobank Coronavirus Self-Test Antibody Study**	21

Table S1: Baseline and resurvey assessment in UK biobank

Data Type	Details	Number of Participants	Date of collection
Baseline questionnaire	Sociodemographic factors, family history, psychosocial factors, local environment, lifestyle, health status, medical history, cognitive function	Whole cohort (baseline)	2006-2010
		20,000 (first repeat assessment)	2012-2013
		100,000 target (imaging visit)	2014-ongoing
		60,000 target (repeat imaging)	2019-ongoing
Baseline physical measures	Blood pressure and heart rate, hand grip strength, anthropometry (including bio-impedance), spirometry, heel bone density, arterial stiffness, hearing, eye exam, 4-lead ECG (at rest and during activity)	Whole cohort (baseline)	2006-2010
		20,000 (first repeat assessment)	2012-2013
		100,000 target (imaging visit)	2014-ongoing
		60,000 target (repeat imaging)	2019-ongoing
Web-based questionnaires<br><br>(~330,000 participants with an email are sent invitations. All other participants can access questionnaires via the participant website)	24hr diet recall (4 occasions)	210,000<br>(127,000 completed all 4 occasions)	2011
	Cognitive function	121,000	2014
	Occupational history	122,000	2015
	Mental health	170,000	2017
	Digestive health	185,000	2017
	Food preferences	195,000	2019-2022
	Pain	180,000	2019-2022
	Cognitive function 2	177,000	2021-2022
	Health and well-being	197,000	2022
	Mental well-being	162,000	2022-2023
Physical activity monitor	Accelerometer data on duration and intensity of physical activity	100,000	2013-2016
		2,500 (repeat measurements)	2018
Imaging assessment	MRI abdominal, brain, and heart; full-body DEXA; carotid ultrasound; 12-lead ECG	100,000 target (imaging visit)	2014-ongoing
		60,000 target (repeat imaging), of which 2,000 are part of the COVID repeat imaging study	2019-ongoing
Cardiac monitor	14 days continual ECG to assess atrial fibrillation	36,000 target	2022-ongoing

Abbreviations: DEXA, dual-energy X-ray absorptiometry; ECG, electrocardiogram; MRI, magnetic resonance imaging.. Detailed information on the data currently available in UK Biobank can be found on the UK Biobank data showcase: https://biobank.ndph.ox.ac.uk/showcase/.

Table S2: Health record linkage in UK biobank

Data Type	Details	Number of Participants	Date of collection
Death registrations	ICD-coded cause-specific mortality	Whole cohort	2006-ongoing
Cancer registrations	ICD-coded cancer diagnoses	Whole cohort	England 1971-ongoing<br>Scotland 1957-ongoing<br>Wales 1971-ongoing
Hospital admissions	ICD-coded diagnoses, and OPCS-coded procedures, from hospital inpatient records, including critical care	Whole cohort	England 1997-ongoing<br>Scotland 1981-ongoing<br>Wales 1999-ongoing
Primary care	Includes Read-coded, BNF and dm+d coded data on diagnoses, prescriptions, and referrals	230,000	England 1938-2017<br>Scotland 1939-2017<br>Wales 1948-2017
Primary care (COVID-19 research only)*	Includes Read-coded, SNOMED CT, dm+d coded data on diagnoses, prescriptions, and referrals	409,000	England 1938-2021
SARS-CoV-2 PCR test results	SARS-CoV-2 PCR test results	Whole cohort	2020-ongoing

Abbreviations: ICD, International Classification of Diseases; OPCS, Office of Population Censuses and Surveys Classification of Interventions and Procedures; PCR, polymerase chain reaction. BNF: British National Formulary; dm+d: Dictionary of Medicines and Devices. *Access for new projects expired June 2022.

Table S3: List of the COVID-19 related publication that used UK Biobank data resources.

1. Armstrong J, Rudkin JK, Allen N, Crook DW, Wilson DJ, Wyllie DH, et al. Dynamic linkage of COVID-19 test results between Public Health England’s Second Generation Surveillance System and UK Biobank. Microbial Genomics. 2020;6(7):mgen000397.

2. Atkins JL, Masoli JAH, Delgado J, Pilling LC, Kuo CL, Kuchel GA, et al. Preexisting Comorbidities Predicting COVID-19 and Mortality in the UK Biobank Community Cohort. J Gerontol A Biol Sci Med Sci. 2020;75(11):2224-30.

3. Aung N, Khanji MY, Munroe PB, Petersen SE. Causal Inference for Genetic Obesity, Cardiometabolic Profile and COVID-19 Susceptibility: A Mendelian Randomization Study. Front Genet. 2020;11:586308.

4. Batty GD, Deary IJ, Luciano M, Altschul DM, Kivimaki M, Gale CR. Psychosocial factors and hospitalisations for COVID-19: Prospective cohort study based on a community sample. Brain Behav Immun. 2020;89:569-78.

5. Chadeau-Hyam M, Bodinier B, Elliott J, Whitaker MD, Tzoulaki I, Vermeulen R, et al. Risk factors for positive and negative COVID-19 tests: a cautious and in-depth analysis of UK biobank data. Int J Epidemiol. 2020;49(5):1454-67.

6. Chudasama YV, Gillies CL, Appiah K, Zaccardi F, Razieh C, Davies MJ, et al. Multimorbidity and SARS-CoV-2 infection in UK Biobank. Diabetes Metab Syndr. 2020;14(5):775-6.

7. Chudasama YV, Zaccardi F, Khunti K. Response to letter to the editor by Abhipsha Sur Roy and Amol Joshi regarding the article: 'Multimorbidity and SARS-CoV-2 infection in UK Biobank' (Chudasama et al.). Diabetes Metab Syndr. 2020;14(5):1281.

8. Curtis D. Variants in ACE2 and TMPRSS2 Genes Are Not Major Determinants of COVID-19 Severity in UK Biobank Subjects. Hum Hered. 2020;85(2):66-8.

9. Denaxas S, Shah AD, Mateen BA, Kuan V, Quint JK, Fitzpatrick N, et al. A semi-supervised approach for rapidly creating clinical biomarker phenotypes in the UK Biobank using different primary care EHR and clinical terminology systems. JAMIA Open. 2020;3(4):545-56.

10. Dubey S, Ramakrishnan V, Naveen P, Dubey A. Rapidly progressive dementia in the COVID-19 era. Alzheimer's & Dementia. 2020;16(S5):e047578.

11. Grant WB, McDonnell SL. Letter in response to the article: Vitamin D concentrations and COVID-19 infection in UK biobank (Hastie et al.). Diabetes Metab Syndr. 2020;14(5):893-4.

12. Griffith GJ, Morris TT, Tudball MJ, Herbert A, Mancano G, Pike L, et al. Collider bias undermines our understanding of COVID-19 disease risk and severity. Nat Commun. 2020;11(1):5749.

13. Hamer M, Gale CR, Batty GD. Diabetes, glycaemic control, and risk of COVID-19 hospitalisation: Population-based, prospective cohort study. Metabolism. 2020;112:154344.

14. Hamer M, Kivimaki M, Gale CR, Batty GD. Lifestyle risk factors, inflammatory mechanisms, and COVID-19 hospitalization: A community-based cohort study of 387,109 adults in UK. Brain Behav Immun. 2020;87:184-7.

15. Hastie CE, Mackay DF, Ho F, Celis-Morales CA, Katikireddi SV, Niedzwiedz CL, et al. Vitamin D concentrations and COVID-19 infection in UK Biobank. Diabetes Metab Syndr. 2020;14(4):561-5.

16. Hastie CE, Mackay DF, Ho F, Celis-Morales CA, Katikireddi SV, Niedzwiedz CL, et al. Corrigendum to "Vitamin D concentrations and COVID-19 infection in UK Biobank" [Diabetes Metabol Syndr: Clin Res Rev 2020 14 (4) 561-5]. Diabetes Metab Syndr. 2020;14(5):1315-6.

17. Ho FK, Celis-Morales CA, Gray SR, Katikireddi SV, Niedzwiedz CL, Hastie C, et al. Modifiable and non-modifiable risk factors for COVID-19, and comparison to risk factors for influenza and pneumonia: results from a UK Biobank prospective cohort study. BMJ Open. 2020;10(11):e040402.

18. Ho FK, Petermann-Rocha F, Gray SR, Jani BD, Katikireddi SV, Niedzwiedz CL, et al. Is older age associated with COVID-19 mortality in the absence of other risk factors? General population cohort study of 470,034 participants. PLoS One. 2020;15(11):e0241824.

19. Kasparian K, Graykowski D, Cudaback E. Commentary: APOE e4 Genotype Predicts Severe COVID-19 in the UK Biobank Community Cohort. Front Immunol. 2020;11:1939.

20. Khanji MY, Aung N, Chahal CAA, Petersen SE. COVID-19 and the UK Biobank-Opportunities and Challenges for Research and Collaboration With Other Large Population Studies. Front Cardiovasc Med. 2020;7:156.

21. Kolin DA, Kulm S, Christos PJ, Elemento O. Clinical, regional, and genetic characteristics of Covid-19 patients from UK Biobank. PLoS One. 2020;15(11):e0241264.

22. Kuo CL, Melzer D. Response to Comment on "ApoE e4e4 Genotype and Mortality With COVID-19 in UK Biobank" by Kuo et al. J Gerontol A Biol Sci Med Sci. 2020;75(11):2235-6.

23. Kuo CL, Pilling LC, Atkins JL, Masoli JAH, Delgado J, Kuchel GA, et al. ApoE e4e4 Genotype and Mortality With COVID-19 in UK Biobank. J Gerontol A Biol Sci Med Sci. 2020;75(9):1801-3.

24. Kuo CL, Pilling LC, Atkins JL, Masoli JAH, Delgado J, Kuchel GA, et al. APOE e4 Genotype Predicts Severe COVID-19 in the UK Biobank Community Cohort. J Gerontol A Biol Sci Med Sci. 2020;75(11):2231-2.

25. Larvin H, Wilmott S, Wu J, Kang J. The Impact of Periodontal Disease on Hospital Admission and Mortality During COVID-19 Pandemic. Front Med (Lausanne). 2020;7:604980.

26. Lassale C, Gaye B, Hamer M, Gale CR, Batty GD. Ethnic disparities in hospitalisation for COVID-19 in England: The role of socioeconomic factors, mental health, and inflammatory and pro-inflammatory factors in a community-based cohort study. Brain Behav Immun. 2020;88:44-9.

27. McQueenie R, Foster HME, Jani BD, Katikireddi SV, Sattar N, Pell JP, et al. Multimorbidity, polypharmacy, and COVID-19 infection within the UK Biobank cohort. PLoS One. 2020;15(8):e0238091.

28. Mulholland RH, Sinha IP. Ethnicity and COVID-19 infection: are the pieces of the puzzle falling into place? BMC Med. 2020;18(1):206.

29. Mutambudzi M, Niedwiedz C, Macdonald EB, Leyland A, Mair F, Anderson J, et al. Occupation and risk of severe COVID-19: prospective cohort study of 120 075 UK Biobank participants. Occup Environ Med. 2020;78(5):307-14.

30. Niedzwiedz CL, O'Donnell CA, Jani BD, Demou E, Ho FK, Celis-Morales C, et al. Ethnic and socioeconomic differences in SARS-CoV-2 infection: prospective cohort study using UK Biobank. BMC Med. 2020;18(1):160.

31. Nikogosov DA, Shevlyakov AD, Baranova AV. Comment on "ApoE e4e4 Genotype and Mortality With COVID-19 in UK Biobank" by Kuo et al. J Gerontol A Biol Sci Med Sci. 2020;75(11):2233-4.

32. Patel AP, Paranjpe MD, Kathiresan NP, Rivas MA, Khera AV. Race, socioeconomic deprivation, and hospitalization for COVID-19 in English participants of a national biobank. Int J Equity Health. 2020;19(1):114.

33. Petermann-Rocha F, Hanlon P, Gray SR, Welsh P, Gill JMR, Foster H, et al. Comparison of two different frailty measurements and risk of hospitalisation or death from COVID-19: findings from UK Biobank. BMC Med. 2020;18(1):355.

34. Raisi-Estabragh Z, McCracken C, Ardissino M, Bethell MS, Cooper J, Cooper C, et al. Renin-Angiotensin-Aldosterone System Blockers Are Not Associated With Coronavirus Disease 2019 (COVID-19) Hospitalization: Study of 1,439 UK Biobank Cases. Front Cardiovasc Med. 2020;7:138.

35. Raisi-Estabragh Z, McCracken C, Bethell MS, Cooper J, Cooper C, Caulfield MJ, et al. Greater risk of severe COVID-19 in Black, Asian and Minority Ethnic populations is not explained by cardiometabolic, socioeconomic or behavioural factors, or by 25(OH)-vitamin D status: study of 1326 cases from the UK Biobank. J Public Health (Oxf). 2020;42(3):451-60.

36. Razieh C, Zaccardi F, Davies MJ, Khunti K, Yates T. Body mass index and the risk of COVID-19 across ethnic groups: Analysis of UK Biobank. Diabetes Obes Metab. 2020;22(10):1953-4.

37. Roy AS, Matson M, Herlekar R. Response to 'Vitamin D concentrations and COVID-19 infection in UK Biobank'. Diabetes Metab Syndr. 2020;14(5):777.

38. Sattar N, Ho FK, Gill JM, Ghouri N, Gray SR, Celis-Morales CA, et al. BMI and future risk for COVID-19 infection and death across sex, age and ethnicity: Preliminary findings from UK biobank. Diabetes Metab Syndr. 2020;14(5):1149-51.

39. Sur Roy A, Joshi A. Response to 'Multimorbidity and SARS-CoV-2 infection in UK biobank'. Diabetes Metab Syndr. 2020;14(5):969.

40. Toh C, Brody JP. Evaluation of a genetic risk score for severity of COVID-19 using human chromosomal-scale length variation. Hum Genomics. 2020;14(1):36.

41. van der Meer D, Pinzon-Espinosa J, Lin BD, Tijdink JK, Vinkers CH, Guloksuz S, et al. Associations between psychiatric disorders, COVID-19 testing probability and COVID-19 testing results: findings from a population-based study. BJPsych Open. 2020;6(5):e87.

42. Woolford SJ, D'Angelo S, Curtis EM, Parsons CM, Ward KA, Dennison EM, et al. COVID-19 and associations with frailty and multimorbidity: a prospective analysis of UK Biobank participants. Aging Clin Exp Res. 2020;32(9):1897-905.

43. Yanai H. Metabolic Syndrome and COVID-19. Cardiol Res. 2020;11(6):360-5.

44. Yang H, Chen W, Hu Y, Chen Y, Zeng Y, Sun Y, et al. Pre-pandemic psychiatric disorders and risk of COVID-19: a UK Biobank cohort analysis. Lancet Healthy Longev. 2020;1(2):e69-e79.

45. Yates T, Razieh C, Zaccardi F, Davies MJ, Khunti K. Obesity and risk of COVID-19: analysis of UK biobank. Prim Care Diabetes. 2020;14(5):566-7.

46. Zhang X, Li X, Sun Z, He Y, Xu W, Campbell H, et al. Physical activity and COVID-19: an observational and Mendelian randomisation study. J Glob Health. 2020;10(2):020514.

47. Zhu Z, Hasegawa K, Ma B, Fujiogi M, Camargo CA, Jr., Liang L. Association of obesity and its genetic predisposition with the risk of severe COVID-19: Analysis of population-based cohort data. Metabolism. 2020;112:154345.

48. Abdallah CG. Brain Networks Associated With COVID-19 Risk: Data From 3662 Participants. Chronic Stress (Thousand Oaks). 2021;5:24705470211066770.

49. Amin HA, Drenos F. No evidence that vitamin D is able to prevent or affect the severity of COVID-19 in individuals with European ancestry: a Mendelian randomisation study of open data. BMJ Nutr Prev Health. 2021;4(1):42-8.

50. Anderson JJ, Ho FK, Niedzwiedz CL, Katikireddi SV, Celis-Morales C, Iliodromiti S, et al. Remote history of VTE is associated with severe COVID-19 in middle and older age: UK Biobank cohort study. J Thromb Haemost. 2021;19(10):2533-8.

51. Batty GD, Deary IJ, Gale CR. Pre-pandemic cognitive function and COVID-19 mortality: prospective cohort study. Eur J Epidemiol. 2021;36(5):559-64.

52. Butler-Laporte G, Nakanishi T, Mooser V, Morrison DR, Abdullah T, Adeleye O, et al. Vitamin D and COVID-19 susceptibility and severity in the COVID-19 Host Genetics Initiative: A Mendelian randomization study. PLoS Med. 2021;18(6):e1003605.

53. Chen HH, Shaw DM, Petty LE, Graff M, Bohlender RJ, Polikowsky HG, et al. Host genetic effects in pneumonia. Am J Hum Genet. 2021;108(1):194-201.

54. Christensen RAG, Arneja J, St Cyr K, Sturrock SL, Brooks JD. The association of estimated cardiorespiratory fitness with COVID-19 incidence and mortality: A cohort study. PLoS One. 2021;16(5):e0250508.

55. Christensen RAG, Sturrock SL, Arneja J, Brooks JD. Measures of Adiposity and Risk of Testing Positive for SARS-CoV-2 in the UK Biobank Study. J Obes. 2021;2021:8837319.

56. Chudasama YV, Zaccardi F, Gillies CL, Razieh C, Yates T, Kloecker DE, et al. Patterns of multimorbidity and risk of severe SARS-CoV-2 infection: an observational study in the U.K. BMC Infect Dis. 2021;21(1):908.

57. Clark S, Lomax N, Morris M, Pontin F, Birkin M. Clustering Accelerometer Activity Patterns from the UK Biobank Cohort. Sensors (Basel). 2021;21(24):8220.

58. Clark S, Morris M, Lomax N, Birkin M. Can a data driven obesity classification system identify those at risk of severe COVID-19 in the UK Biobank cohort study? Int J Obes (Lond). 2021;45(10):2281-5.

59. Dabbah MA, Reed AB, Booth ATC, Yassaee A, Despotovic A, Klasmer B, et al. Machine learning approach to dynamic risk modeling of mortality in COVID-19: a UK Biobank study. Sci Rep. 2021;11(1):16936.

60. Dai XJ, Tan L, Ren L, Shao Y, Tao W, Wang Y. COVID-19 Risk Appears to Vary Across Different Alcoholic Beverages. Front Nutr. 2021;8:772700.

61. Darling AL, Ahmadi KR, Ward KA, Harvey NC, Alves AC, Dunn-Walters DK, et al. Vitamin D concentration, body mass index, ethnicity and SARS-CoV-2/COVID-19: initial analysis of the first- reported UK Biobank Cohort positive cases (n 1474) compared with negative controls (n 4643). Proceedings of the Nutrition Society. 2021;80(Oce1):e17.

62. Davies G, Mazess RB, Benskin LL. Letter to the editor in response to the article: "Vitamin D concentrations and COVID-19 infection in UK biobank" (Hastie et al.). Diabetes Metab Syndr. 2021;15(2):643-4.

63. Davies NG, Barnard RC, Jarvis CI, Russell TW, Semple MG, Jit M, et al. Association of tiered restrictions and a second lockdown with COVID-19 deaths and hospital admissions in England: a modelling study. Lancet Infect Dis. 2021;21(4):482-92.

64. de Jong M, Woodward M, Peters SAE. Diabetes and COVID-19-Related Mortality in Women and Men in the UK Biobank: Comparisons With Influenza/Pneumonia and Coronary Heart Disease. Diabetes Care. 2021;44(2):e22-e4.

65. Dey S, Bose A, Saha S, Chakraborty P, Ghalwash M, Guzm XENSA, et al. Impact of Clinical and Genomic Factors on COVID-19 Disease Severity. AMIA Annu Symp Proc. 2021;2021:378-87.

66. Didikoglu A, Maharani A, Pendleton N, Canal MM, Payton A. Early life factors and COVID-19 infection in England: A prospective analysis of UK Biobank participants. Early Hum Dev. 2021;155:105326.

67. Dite GS, Murphy NM, Allman R. Development and validation of a clinical and genetic model for predicting risk of severe COVID-19. Epidemiol Infect. 2021;149:e162.

68. Dite GS, Murphy NM, Allman R. An integrated clinical and genetic model for predicting risk of severe COVID-19: A population-based case-control study. PLoS One. 2021;16(2):e0247205.

69. Drozd M, Pujades-Rodriguez M, Lillie PJ, Straw S, Morgan AW, Kearney MT, et al. Non-communicable disease, sociodemographic factors, and risk of death from infection: a UK Biobank observational cohort study. Lancet Infect Dis. 2021;21(8):1184-91.

70. Elliott J, Bodinier B, Whitaker M, Delpierre C, Vermeulen R, Tzoulaki I, et al. COVID-19 mortality in the UK Biobank cohort: revisiting and evaluating risk factors. Eur J Epidemiol. 2021;36(3):299-309.

71. Elliott J, Bodinier B, Whitaker M, Tzoulaki I, Elliott P, Chadeau-Hyam M. Abstract 041: Cardiovascular Disease, Hypertension, Diabetes And Cystatin C Jointly Predict Covid-19 Mortality Alongside Age, Male Sex And Black Ethnicity. Circulation. 2021;143(Suppl_1).

72. Fan X, Liu Z, Miyata T, Dasarathy S, Rotroff DM, Wu X, et al. Effect of Acid Suppressants on the Risk of COVID-19: A Propensity Score-Matched Study Using UK Biobank. Gastroenterology. 2021;160(1):455-8 e5.

73. Fan X, Liu Z, Poulsen KL, Wu X, Miyata T, Dasarathy S, et al. Alcohol Consumption Is Associated with Poor Prognosis in Obese Patients with COVID-19: A Mendelian Randomization Study Using UK Biobank. Nutrients. 2021;13(5):1592.

74. Fan X, Yin C, Wang J, Yang M, Ma H, Jin G, et al. Pre-diagnostic circulating concentrations of insulin-like growth factor-1 and risk of COVID-19 mortality: results from UK Biobank. Eur J Epidemiol. 2021;36(3):311-8.

75. Fatima Y, Bucks RS, Mamun AA, Skinner I, Rosenzweig I, Leschziner G, et al. Shift work is associated with increased risk of COVID-19: Findings from the UK Biobank cohort. J Sleep Res. 2021;30(5):e13326.

76. Fernández-Sanlés A, Carter A, Millard L, Smith D, Griffith G, Clayton G, et al. 1484Selection bias in COVID-19 research: Prospective analyses of two UK cohort studies. International Journal of Epidemiology. 2021;50(Supplement_1):dyab168.442.

77. Freuer D, Linseisen J, Meisinger C. Impact of body composition on COVID-19 susceptibility and severity: A two-sample multivariable Mendelian randomization study. Metabolism. 2021;118:154732.

78. Ghodsian N, Abner E, Gobeil É, Taba N, Amand AS, Perrot N, et al. Electronic Health Record-Based Genome-Wide Meta-Analysis Identifies New Susceptibility Loci for Non-Alcoholic Fatty Liver Disease. Journal of the Endocrine Society. 2021;5(Supplement_1):A501-A.

79. Gkouskou K, Vasilogiannakopoulou T, Andreakos E, Davanos N, Gazouli M, Sanoudou D, et al. COVID-19 enters the expanding network of apolipoprotein E4-related pathologies. Redox Biol. 2021;41:101938.

80. Goo T, Han K, Apio C, Park T. Analysis of COVID-19 genetic risk susceptibility using UK Biobank SNP genotype data. International Journal of Data Mining and Bioinformatics. 2021;25(1-2):1-16.

81. Griffanti L, Raman B, Alfaro-Almagro F, Filippini N, Cassar MP, Sheerin F, et al. Adapting the UK Biobank Brain Imaging Protocol and Analysis Pipeline for the C-MORE Multi-Organ Study of COVID-19 Survivors. Front Neurol. 2021;12:753284.

82. Hamet P, Pausova Z, Attaoua R, Hishmih C, Haloui M, Shin J, et al. SARS-CoV-2 Receptor ACE2 Gene Is Associated with Hypertension and Severity of COVID 19: Interaction with Sex, Obesity, and Smoking. Am J Hypertens. 2021;34(4):367-76.

83. Hamrouni M, Roberts MJ, Thackray A, Stensel DJ, Bishop N. Associations of obesity, physical activity level, inflammation and cardiometabolic health with COVID-19 mortality: a prospective analysis of the UK Biobank cohort. BMJ Open. 2021;11(11):e055003.

84. Hastie CE, Pell JP, Sattar N. Vitamin D and COVID-19 infection and mortality in UK Biobank. Eur J Nutr. 2021;60(1):545-8.

85. Henne SK, Hochfeld LM, Maj C, Nothen MM, Heilmann-Heimbach S. Systematic investigation of a potential epidemiological and genetic association between male androgenetic alopecia and COVID-19. Skin Health Dis. 2021;1(4):e72.

86. Hilser JR, Han Y, Biswas S, Gukasyan J, Cai Z, Zhu R, et al. Association of serum HDL-cholesterol and apolipoprotein A1 levels with risk of severe SARS-CoV-2 infection. J Lipid Res. 2021;62:100061.

87. Hou C, Hu Y, Yang H, Chen W, Zeng Y, Ying Z, et al. COVID-19 and risk of subsequent life-threatening secondary infections: a matched cohort study in UK Biobank. BMC Med. 2021;19(1):301.

88. Hu J, Li C, Wang S, Li T, Zhang H. Genetic variants are identified to increase risk of COVID-19 related mortality from UK Biobank data. Hum Genomics. 2021;15(1):10.

89. Jani BD, Nicholl BI, Hanlon P, Mair FS, Gill JM, Gray SR, et al. Family history of diabetes and risk of SARS-COV-2 in UK Biobank: A prospective cohort study. Endocrinol Diabetes Metab. 2021;4(4):e00283.

90. Julkunen H, Cichonska A, Slagboom PE, Wurtz P, Nightingale Health UKBI. Metabolic biomarker profiling for identification of susceptibility to severe pneumonia and COVID-19 in the general population. Elife. 2021;10:e63033.

91. Konuma T, Ogawa K, Okada Y. Integration of genetically regulated gene expression and pharmacological library provides therapeutic drug candidates. Hum Mol Genet. 2021;30(3-4):294-304.

92. Kuo CL, Pilling LC, Atkins JL, Masoli JAH, Delgado J, Tignanelli C, et al. Biological Aging Predicts Vulnerability to COVID-19 Severity in UK Biobank Participants. J Gerontol A Biol Sci Med Sci. 2021;76(8):e133-e41.

93. Larvin H, Wilmott S, Kang J, Aggarwal VR, Pavitt S, Wu J. Additive Effect of Periodontal Disease and Obesity on COVID-19 Outcomes. J Dent Res. 2021;100(11):1228-35.

94. Lassale C, Hamer M, Hernaez A, Gale CR, Batty GD. Association of pre-pandemic high-density lipoprotein cholesterol with risk of COVID-19 hospitalisation and death: The UK Biobank cohort study. Prev Med Rep. 2021;23:101461.

95. Lee SF, Niksic M, Rachet B, Sanchez MJ, Luque-Fernandez MA. Socioeconomic Inequalities and Ethnicity Are Associated with a Positive COVID-19 Test among Cancer Patients in the UK Biobank Cohort. Cancers (Basel). 2021;13(7):1514.

96. Lehrer S, Rheinstein P. Eyeglasses Reduce Risk of COVID-19 Infection. In Vivo. 2021;35(3):1581-2.

97. Lehrer S, Rheinstein PH. Homozygosity for rs17775810 Minor Allele Associated With Reduced Mortality of COVID-19 in the UK Biobank Cohort. In Vivo. 2021;35(2):965-8.

98. Lehrer S, Rheinstein PH. ABO blood groups, COVID-19 infection and mortality. Blood Cells Mol Dis. 2021;89:102571.

99. Lehrer S, Rheinstein PH. BIN1 rs744373 SNP and COVID-19 mortality. World Acad Sci J. 2021;3(2):13.

100. Lehrer S, Rheinstein PH. Common drugs, vitamins, nutritional supplements and COVID-19 mortality. Int J Funct Nutr. 2021;2(1):4.

101. Leong A, Cole JB, Brenner LN, Meigs JB, Florez JC, Mercader JM. Cardiometabolic risk factors for COVID-19 susceptibility and severity: A Mendelian randomization analysis. PLoS Med. 2021;18(3):e1003553.

102. Li H, Baldwin E, Zhang X, Kenost C, Luo W, Calhoun EA, et al. Comparison and impact of COVID-19 for patients with cancer: a survival analysis of fatality rate controlling for age, sex and cancer type. BMJ Health Care Inform. 2021;28(1):e100341.

103. Li S, Cao Z, Yang H, Zhang Y, Xu F, Wang Y. Metabolic Healthy Obesity, Vitamin D Status, and Risk of COVID-19. Aging Dis. 2021;12(1):61-71.

104. Li X, van Geffen J, van Weele M, Zhang X, He Y, Meng X, et al. An observational and Mendelian randomisation study on vitamin D and COVID-19 risk in UK Biobank. Sci Rep. 2021;11(1):18262.

105. Lipsitz L, Baker T. 2020 Editor's Choice Articles from Jgms: Focus on Vulnerable Populations. Innovation in Aging. 2021;5(Suppl 1):341-2.

106. Lodge CJ, Doherty A, Bui DS, Cassim R, Lowe AJ, Agusti A, et al. Is asthma associated with COVID-19 infection? A UK Biobank analysis. ERJ Open Res. 2021;7(4):00309-2021.

107. Ma H, Zhou T, Heianza Y, Qi L. Habitual use of vitamin D supplements and risk of coronavirus disease 2019 (COVID-19) infection: a prospective study in UK Biobank. Am J Clin Nutr. 2021;113(5):1275-81.

108. Ma Y, Zhang Y, Li S, Yang H, Li H, Cao Z, et al. Sex Differences in Association Between Anti-Hypertensive Medications and Risk of COVID-19 in Middle-Aged and Older Adults. Drugs Aging. 2021;38(10):921-30.

109. Maidstone R, Anderson SG, Ray DW, Rutter MK, Durrington HJ, Blaikley JF. Shift work is associated with positive COVID-19 status in hospitalised patients. Thorax. 2021;76(6):601-6.

110. Masoli J, Atkins J, Delgado J, Pilling L, Melzer D. 12 Being Non-Frail and Free From Cardiovascular Disease Reduces COVID-19 Risk in 269,164 Older UK Biobank Participants. Age and Ageing. 2021;50(Supplement_1):i1-i6.

111. Masoli J, Kuo CL, Atkins J, Pilling L, Delgado J, Kuchel G, et al. 38 Dementia, Apoe and COVID-19 Severity. Age and Ageing. 2021;50(Supplement_1):i7-i11.

112. McQueenie R, Foster HME, Jani BD, Katikireddi SV, Sattar N, Pell JP, et al. Correction: Multimorbidity, polypharmacy, and COVID-19 infection within the UK Biobank cohort. PLoS One. 2021;16(5):e0251613.

113. Monereo-Sanchez J, Luykx JJ, Pinzon-Espinosa J, Richard G, Motazedi E, Westlye LT, et al. Diphtheria And Tetanus Vaccination History Is Associated With Lower Odds of COVID-19 Hospitalization. Front Immunol. 2021;12:749264.

114. Morys F, Dagher A. Poor Metabolic Health Increases COVID-19-Related Mortality in the UK Biobank Sample. Front Endocrinol (Lausanne). 2021;12:652765.

115. Nalini M, Khoshnia M, Kamangar F, Sharafkhah M, Poustchi H, Pourshams A, et al. Corrigendum to: Joint effect of diabetes and opiate use on all-cause and cause-specific mortality: the Golestan cohort study. Int J Epidemiol. 2021;50(1):353.

116. Papadopoulou A, Musa H, Sivaganesan M, McCoy D, Deloukas P, Marouli E. COVID-19 susceptibility variants associate with blood clots, thrombophlebitis and circulatory diseases. PLoS One. 2021;16(9):e0256988.

117. Patchen B, Clark A, Gaddis N, Hancock D, Cassano P. Genetically Predicted Serum Vitamin D and COVID-19: A Mendelian Randomization Study. Current Developments in Nutrition. 2021;5(Supplement_2):1080-.

118. Patchen BK, Clark AG, Gaddis N, Hancock DB, Cassano PA. Genetically predicted serum vitamin D and COVID-19: a Mendelian randomisation study. BMJ Nutr Prev Health. 2021;4(1):213-25.

119. Patel KHK, Li X, Quint JK, Ware JS, Peters NS, Ng FS. Increasing adiposity and the presence of cardiometabolic morbidity is associated with increased Covid-19-related mortality: results from the UK Biobank. BMC Endocr Disord. 2021;21(1):144.

120. Pathak GA, Singh K, Miller-Fleming TW, Wendt FR, Ehsan N, Hou K, et al. Integrative genomic analyses identify susceptibility genes underlying COVID-19 hospitalization. Nat Commun. 2021;12(1):4569.

121. Pell JP, Hastie CE, Sattar N. Reply to letter of Davie et al. regarding the article: "Vitamin D concentrations and COVID-19 infection in UK Biobank" (Hastie et al.). Diabetes Metab Syndr. 2021;15(2):642.

122. Peters SAE, MacMahon S, Woodward M. Obesity as a risk factor for COVID-19 mortality in women and men in the UK biobank: Comparisons with influenza/pneumonia and coronary heart disease. Diabetes Obes Metab. 2021;23(1):258-62.

123. Prats-Uribe A, Xie J, Prieto-Alhambra D, Petersen I. Smoking and COVID-19 Infection and Related Mortality: A Prospective Cohort Analysis of UK Biobank Data. Clin Epidemiol. 2021;13:357-65.

124. Raisi-Estabragh Z, McCracken C, Cooper J, Fung K, Paiva JM, Khanji MY, et al. Adverse cardiovascular magnetic resonance phenotypes are associated with greater likelihood of incident coronavirus disease 2019: findings from the UK Biobank. Aging Clin Exp Res. 2021;33(4):1133-44.

125. Rauseo E, Lockhart L, Paiva JM, Fung K, Khanji MY, Raisi-Estabragh Z, et al. Automated myocardial segmentation in native t1-mapping cardiovascular magnetic resonance images based on machine learning: a validation study in the UK biobank"s covid-19 subset. European Heart Journal - Cardiovascular Imaging. 2021;22(Supplement_2):jeab090.25.

126. Razieh C, Zaccardi F, Islam N, Gillies CL, Y VC, Rowlands A, et al. Ethnic minorities and COVID-19: examining whether excess risk is mediated through deprivation. Eur J Public Health. 2021;31(3):630-4.

127. Roca-Fernandez A, Dennis A, Nicholls R, McGonigle J, Kelly M, Banerjee R, et al. Hepatic Steatosis, Rather Than Underlying Obesity, Increases the Risk of Infection and Hospitalization for COVID-19. Front Med (Lausanne). 2021;8:636637.

128. Rowlands AV, Dempsey PC, Gillies C, Kloecker DE, Razieh C, Chudasama Y, et al. Association Between Accelerometer-Assessed Physical Activity and Severity of COVID-19 in UK Biobank. Mayo Clin Proc Innov Qual Outcomes. 2021;5(6):997-1007.

129. Rowlands AV, Gillies C, Chudasama Y, Davies MJ, Islam N, Kloecker DE, et al. Association of working shifts, inside and outside of healthcare, with severe COVID-19: an observational study. BMC Public Health. 2021;21(1):773.

130. Rowlands AV, Kloecker DE, Chudasama Y, Davies MJ, Dawkins NP, Edwardson CL, et al. Association of Timing and Balance of Physical Activity and Rest/Sleep With Risk of COVID-19: A UK Biobank Study. Mayo Clin Proc. 2021;96(1):156-64.

131. Scalsky RJ, Chen YJ, Desai K, O'Connell JR, Perry JA, Hong CC. Baseline cardiometabolic profiles and SARS-CoV-2 infection in the UK Biobank. PLoS One. 2021;16(4):e0248602.

132. Sengupta S, Brooks TG, Grant GR, FitzGerald GA. Accounting for Time: Circadian Rhythms in the Time of COVID-19. J Biol Rhythms. 2021;36(1):4-8.

133. Stevens HP, Canovas R, Peter K, Tran H, Kaplan Z, McFadyen JD. Inherited Thrombophilias Are Associated with an Increased Risk of COVID-19 Associated Venous Thromboembolism: A Prospective Population-Based Cohort Study. Blood. 2021;138(Supplement 1):3214-.

134. Tahira AC, Verjovski-Almeida S, Ferreira ST. Dementia is an age-independent risk factor for severity and death in COVID-19 inpatients. Alzheimers Dement. 2021;17(11):1818-31.

135. Topless RK, Phipps-Green A, Leask M, Dalbeth N, Stamp LK, Robinson PC, et al. Gout, Rheumatoid Arthritis, and the Risk of Death Related to Coronavirus Disease 2019: An Analysis of the UK Biobank. ACR Open Rheumatol. 2021;3(5):333-40.

136. Travaglio M, Yu Y, Popovic R, Selley L, Leal NS, Martins LM. Links between air pollution and COVID-19 in England. Environ Pollut. 2021;268(Pt A):115859.

137. Trivedi A, Liles WC, Becker N, Egan C, Ferres JL, Lee A, et al. Parameters of Chronic Kidney Disease to Identify Outpatients at Increased Risk for COVID-19 Mortality: A Cohort Study of UK Biobank Participants. The Open Urology & Nephrology Journal. 2021;14(1):26-8.

138. van Moorsel CHM, van der Vis JJ, Duckworth A, Scotton CJ, Benschop C, Ellinghaus D, et al. The MUC5B Promoter Polymorphism Associates With Severe COVID-19 in the European Population. Front Med (Lausanne). 2021;8:668024.

139. Veronese N, Smith L, Barbagallo M, Giannelli G, Caruso MG, Cisternino AM, et al. Neurological diseases and COVID-19: prospective analyses using the UK Biobank. Acta Neurol Belg. 2021;121(5):1295-303.

140. Vu TT, Rydland KJ, Achenbach CJ, Van Horn L, Cornelis MC. Dietary Behaviors and Incident COVID-19 in the UK Biobank. Nutrients. 2021;13(6):2114.

141. Wang J, Zhu J, Yang H, Hu Y, Sun Y, Ying Z, et al. Cardiovascular-related deaths at the beginning of the COVID-19 outbreak: a prospective analysis based on the UK Biobank. BMJ Open. 2021;11(6):e046931.

142. Wang L, Jackson VE, Fearnley LG, Bahlo M. UKB.COVID19: an R package for UK Biobank COVID-19 data processing and analysis. F1000Research. 2021;10:830.

143. Wang Q, Codd V, Raisi-Estabragh Z, Musicha C, Bountziouka V, Kaptoge S, et al. Shorter leukocyte telomere length is associated with adverse COVID-19 outcomes: A cohort study in UK Biobank. EBioMedicine. 2021;70:103485.

144. Wang Y, Yang Y, Ren L, Shao Y, Tao W, Dai XJ. Preexisting Mental Disorders Increase the Risk of COVID-19 Infection and Associated Mortality. Front Public Health. 2021;9:684112.

145. Westerman KE, Lin J, Sevilla-Gonzalez MDR, Tadess B, Marchek C, Manning AK. Gene-Environment Interaction Analysis Incorporating Sex, Cardiometabolic Diseases, and Multiple Deprivation Index Reveals Novel Genetic Associations With COVID-19 Severity. Front Genet. 2021;12:782172.

146. Wong KC, Xiang Y, Yin L, So HC. Uncovering Clinical Risk Factors and Predicting Severe COVID-19 Cases Using UK Biobank Data: Machine Learning Approach. JMIR Public Health Surveill. 2021;7(9):e29544.

147. Woodward M, Peters SAE, Harris K. Social deprivation as a risk factor for COVID-19 mortality among women and men in the UK Biobank: nature of risk and context suggests that social interventions are essential to mitigate the effects of future pandemics. J Epidemiol Community Health. 2021;75(11):1050-5.

148. Woolford SJ, D’Angelo S, Curtis EM, Parsons CM, Ward KA, Dennison EM, et al. 84 COVID-19 and Associations with Frailty and Multimorbidity: A Prospective Analysis of UK Biobank Participants. Age and Ageing. 2021;50(Supplement_1):i12-i42.

149. Xiang Y, Wong KC, So HC. Exploring Drugs and Vaccines Associated with Altered Risks and Severity of COVID-19: A UK Biobank Cohort Study of All ATC Level-4 Drug Categories Reveals Repositioning Opportunities. Pharmaceutics. 2021;13(9):1514.

150. Yates T, Razieh C, Zaccardi F, Rowlands AV, Seidu S, Davies MJ, et al. Obesity, walking pace and risk of severe COVID-19 and mortality: analysis of UK Biobank. Int J Obes (Lond). 2021;45(5):1155-9.

151. Ying K, Zhai R, Pyrkov TV, Shindyapina AV, Mariotti M, Fedichev PO, et al. Genetic and phenotypic analysis of the causal relationship between aging and COVID-19. Commun Med (Lond). 2021;1(1):35.

152. Yoshikawa M, Asaba K, Nakayama T. Estimating causal effects of atherogenic lipid-related traits on COVID-19 susceptibility and severity using a two-sample Mendelian randomization approach. BMC Med Genomics. 2021;14(1):269.

153. Yu Y, Travaglio M, Popovic R, Leal NS, Martins LM. Alzheimer's and Parkinson's Diseases Predict Different COVID-19 Outcomes: A UK Biobank Study. Geriatrics (Basel). 2021;6(1):10.

154. Zhang J, Pellicori P, Schutte R, Cleland JG. The association between blood groups and COVID-19 infection: a study from the UK Biobank. J Intern Med. 2021;289(5):747-8.

155. Zhang Y, Yang H, Li S, Li WD, Wang J, Wang Y. Association analysis framework of genetic and exposure risks for COVID-19 in middle-aged and elderly adults. Mech Ageing Dev. 2021;194:111433.

156. Zheng X, Ulsa MC, Li P, Gao L, Hu K. 699 Sleep Health Traits and COVID-19: Mortality Risk from the UK Biobank. Sleep. 2021;44(Supplement_2):A273-A.

157. Zhou J, Liu C, Sun Y, Huang W, Ye K. Cognitive disorders associated with hospitalization of COVID-19: Results from an observational cohort study. Brain Behav Immun. 2021;91:383-92.

158. Zhou J, Sun Y, Huang W, Ye K. Altered Blood Cell Traits Underlie a Major Genetic Locus of Severe COVID-19. J Gerontol A Biol Sci Med Sci. 2021;76(8):e147-e54.

159. Correction: Occupation and risk of severe COVID-19: prospective cohort study of 120 075 UK Biobank participants. Occup Environ Med. 2022;79(2):e3.

160. Allen D, Vergara-Lope A, Wilson D. Structural health determinants and comorbidity risk factors for COVID-19 outcomes among participants in the UK Biobank. International Journal of Population Data Science. 2022;7(3):1836.

161. Au Yeung SL, Li AM, He B, Kwok KO, Schooling CM. Association of smoking, lung function and COPD in COVID-19 risk: a two-step Mendelian randomization study. Addiction. 2022;117(7):2027-36.

162. Azizi Z, Shiba Y, Alipour P, Maleki F, Raparelli V, Norris C, et al. Importance of sex and gender factors for COVID-19 infection and hospitalisation: a sex-stratified analysis using machine learning in UK Biobank data. BMJ Open. 2022;12(5):e050450.

163. Batty GD, Gaye B, Gale CR, Hamer M, Lassale C. Explaining Ethnic Differentials in COVID-19 Mortality: A Cohort Study. Am J Epidemiol. 2022;191(2):275-81.

164. Both C, Abdelhakim S, Acosta J, Szejko N, Leasure AC, Jasne A, et al. Abstract TMP22: Genetic Predisposition To Cardiovascular Disease Is Associated With Higher Risk Of Stroke In Persons With COVID-19. Stroke. 2022;53(Suppl_1):atmp22-atmp.

165. Boucher BJ. Vitamin D deficiency in British South Asians, a persistent but avoidable problem associated with many health risks (including rickets, T2DM, CVD, COVID-19 and pregnancy complications): the case for correcting this deficiency. Endocr Connect. 2022;11(12):e220234.

166. Chen W, Zeng Y, Suo C, Yang H, Chen Y, Hou C, et al. Genetic predispositions to psychiatric disorders and the risk of COVID-19. BMC Med. 2022;20(1):314.

167. Clift AK, von Ende A, Tan PS, Sallis HM, Lindson N, Coupland CAC, et al. Smoking and COVID-19 outcomes: an observational and Mendelian randomisation study using the UK Biobank cohort. Thorax. 2022;77(1):65-73.

168. Coleman P, Barber TM, van Rens T, Hanson P, Coffey A, Oyebode O. COVID-19 Outcomes in Minority Ethnic Groups: Do Obesity and Metabolic Risk Play a Role? Curr Obes Rep. 2022;11(3):107-15.

169. Crossfield SSR, Chaddock NJM, Iles MM, Pujades-Rodriguez M, Morgan AW. Interplay between demographic, clinical and polygenic risk factors for severe COVID-19. Int J Epidemiol. 2022;51(5):1384-95.

170. Das S, Taylor K, Kozubek J, Sardell J, Gardner S. Genetic risk factors for ME/CFS identified using combinatorial analysis. J Transl Med. 2022;20(1):598.

171. Devereux-Cooke A, Leary S, McGrath SJ, Northwood E, Redshaw A, Shepherd C, et al. DecodeME: community recruitment for a large genetics study of myalgic encephalomyelitis / chronic fatigue syndrome. BMC Neurol. 2022;22(1):269.

172. Di Maio S, Lamina C, Coassin S, Forer L, Wurzner R, Schonherr S, et al. Lipoprotein(a) and SARS-CoV-2 infections: Susceptibility to infections, ischemic heart disease and thromboembolic events. J Intern Med. 2022;291(1):101-7.

173. Douaud G, Lee S, Alfaro-Almagro F, Arthofer C, Wang C, McCarthy P, et al. SARS-CoV-2 is associated with changes in brain structure in UK Biobank. Nature. 2022;604(7907):697-707.

174. Duff E, Zelaya F, Almagro FA, Miller KL, Martin N, Nichols TE, et al. Reliability of multi-site UK Biobank MRI brain phenotypes for the assessment of neuropsychiatric complications of SARS-CoV-2 infection: The COVID-CNS travelling heads study. PLoS One. 2022;17(9):e0273704.

175. Foster HME, Ho FK, Mair FS, Jani BD, Sattar N, Katikireddi SV, et al. The association between a lifestyle score, socioeconomic status, and COVID-19 outcomes within the UK Biobank cohort. BMC Infect Dis. 2022;22(1):273.

176. Freitas RS, Roque CR, Matos GA, Belayev L, de Azevedo OGR, Alvarez-Leite JI, et al. Immunoinflammatory role of apolipoprotein E4 in malnutrition and enteric infections and the increased risk for chronic diseases under adverse environments. Nutr Rev. 2022;80(5):1001-12.

177. Gao M, Wang Q, Piernas C, Astbury NM, Jebb SA, Holmes MV, et al. Associations between body composition, fat distribution and metabolic consequences of excess adiposity with severe COVID-19 outcomes: observational study and Mendelian randomisation analysis. Int J Obes (Lond). 2022;46(5):943-50.

178. Gillies CL, Rowlands AV, Razieh C, Nafilyan V, Chudasama Y, Islam N, et al. Association between household size and COVID-19: A UK Biobank observational study. J R Soc Med. 2022;115(4):138-44.

179. Hamrouni M, Roberts MJ, Bishop NC. The joint associations of physical activity and TV viewing time with COVID-19 mortality: An analysis of UK Biobank. J Sports Sci. 2022:1-8.

180. Hamrouni M, Roberts MJ, Bishop NC. Television Viewing Time, Overweight, Obesity, and Severe COVID-19: A Brief Report From UK Biobank. J Phys Act Health. 2022;19(12):837-41.

181. Hassan L, Firth J. COVID-19 infection, hospitalisation and mortality rates in people with severe mental illness: findings from two UK cohort studies. European Psychiatry. 2022;65(S1):S75-S6.

182. Hassan L, Peek N, Lovell K, Carvalho AF, Solmi M, Stubbs B, et al. Disparities in COVID-19 infection, hospitalisation and death in people with schizophrenia, bipolar disorder, and major depressive disorder: a cohort study of the UK Biobank. Mol Psychiatry. 2022;27(2):1248-55.

183. Howarth S, Quinton R. Missed opportunities in the treatment of Turner syndrome: a case discussion and review of the guidelines. BMJ Case Rep. 2022;15(8):e250870.

184. Hu Y, Yang H, Hou C, Chen W, Zhang H, Ying Z, et al. COVID-19 related outcomes among individuals with neurodegenerative diseases: a cohort analysis in the UK biobank. BMC Neurol. 2022;22(1):15.

185. Huang BH, Inan-Eroglu E, Shaban RZ, Hamer M, Britton A, Stamatakis E. Alcohol intake and mortality risk of COVID-19, pneumonia, and other infectious diseases: An analysis of 437191 UK biobank participants. Prev Med Rep. 2022;26:101751.

186. Huang D, Xu R, Na R. Cannabis Use Is Associated With Lower COVID-19 Susceptibility but Poorer Survival. Front Public Health. 2022;10:829715.

187. Huang QM, Zhang PD, Li ZH, Zhou JM, Liu D, Zhang XR, et al. Genetic Risk and Chronic Obstructive Pulmonary Disease Independently Predict the Risk of Incident Severe COVID-19. Ann Am Thorac Soc. 2022;19(1):58-65.

188. Hui LL, Nelson EAS, Lin SL, Zhao JV. The role of vitamin C in pneumonia and COVID-19 infection in adults with European ancestry: a Mendelian randomisation study. Eur J Clin Nutr. 2022;76(4):588-91.

189. Kang J, Jia T, Jiao Z, Shen C, Xie C, Cheng W, et al. Increased brain volume from higher cereal and lower coffee intake: shared genetic determinants and impacts on cognition and metabolism. Cereb Cortex. 2022;32(22):5163-74.

190. Kessler MD, Damask A, O'Keeffe S, Banerjee N, Li D, Watanabe K, et al. Common and rare variant associations with clonal haematopoiesis phenotypes. Nature. 2022;612(7939):301-9.

191. Kunnath Ramachandran A, Das S, Shenoy GG, Mudgal J, Joseph A. Relation between apolipoprotein e in alzheimer's disease and sars-cov-2 and their treatment strategy: A review. CNS Neurol Disord Drug Targets. 2022;22.

192. Li J, Tian A, Yang D, Zhang M, Chen L, Wen J, et al. Celiac Disease and the Susceptibility of COVID-19 and the Risk of Severe COVID-19: A Mendelian Randomization Study. Clin Transl Gastroenterol. 2022;13(5):e00480.

193. Li J, Tian A, Zhu H, Chen L, Wen J, Liu W, et al. Mendelian Randomization Analysis Reveals No Causal Relationship Between Nonalcoholic Fatty Liver Disease and Severe COVID-19. Clin Gastroenterol Hepatol. 2022;20(7):1553-60 e78.

194. Lin LY, Mulick A, Mathur R, Smeeth L, Warren-Gash C, Langan SM. The association between vitamin D status and COVID-19 in England: A cohort study using UK Biobank. PLoS One. 2022;17(6):e0269064.

195. Lin Y, Ma B, Yang Y, Chen Y, Huang J, Li W, et al. Impaired kidney function biomarkers and risk of severe COVID-19: Analysis of population-based cohort data. Mol Genet Genomic Med. 2022;10(11):e2047.

196. Liu Z, Luo Y, Su Y, Wei Z, Li R, He L, et al. Associations of sleep and circadian phenotypes with COVID-19 susceptibility and hospitalization: an observational cohort study based on the UK Biobank and a two-sample Mendelian randomization study. Sleep. 2022;45(6):zsac003.

197. Luo YS, Luo L, Li W, Chen Y, Wu GF, Chen F, et al. Evaluation of a Functional Single Nucleotide Polymorphism of the SARS-CoV-2 Receptor ACE2 That Is Potentially Involved in Long COVID. Front Genet. 2022;13:931562.

198. McManus JM, Sabharwal N, Bazeley P, Sharifi N. Inheritance of a common androgen synthesis variant allele is associated with female COVID susceptibility in UK Biobank. Eur J Endocrinol. 2022;187(1):1-14.

199. Meinlschmidt G, Guemghar S, Roemmel N, Battegay E, Hunziker S, Schaefert R. Depressive symptoms, but not anxiety, predict subsequent diagnosis of Coronavirus disease 19: a national cohort study. Epidemiol Psychiatr Sci. 2022;31:e16.

200. Millard LAC, Fernandez-Sanles A, Carter AR, Hughes RA, Tilling K, Morris TP, et al. Exploring the impact of selection bias in observational studies of COVID-19: a simulation study. Int J Epidemiol. 2022.

201. Obesity JO. Retracted: Measures of Adiposity and Risk of Testing Positive for SARS-CoV-2 in the UK Biobank Study. J Obes. 2022;2022:9848453.

202. Ostendorf BN, Patel MA, Bilanovic J, Hoffmann HH, Carrasco SE, Rice CM, et al. Common human genetic variants of APOE impact murine COVID-19 mortality. Nature. 2022;611(7935):346-51.

203. Paik H, Kim J, Seo S. Analysis of the docking property of host variants of hACE2 for SARS-CoV-2 in a large cohort. PLoS Comput Biol. 2022;18(7):e1009834.

204. Papez V, Moinat M, Voss EA, Bazakou S, Van Winzum A, Peviani A, et al. Transforming and evaluating the UK Biobank to the OMOP Common Data Model for COVID-19 research and beyond. J Am Med Inform Assoc. 2022;30(1):103-11.

205. Pavey H, Kulkarni S, Wood A, Ben-Shlomo Y, Sever P, McEniery C, et al. Primary hypertension, anti-hypertensive medications and the risk of severe COVID-19 in UK Biobank. PLoS One. 2022;17(11):e0276781.

206. Raisi-Estabragh Z, Cooper J, Salih A, Raman B, Lee AM, Neubauer S, et al. Cardiovascular disease and mortality sequelae of COVID-19 in the UK Biobank. Heart. 2022;109(2):119-26.

207. Reeves J, Kooner JS, Zhang W. Accelerated ageing is associated with increased COVID-19 severity and differences across ethnic groups may exist. Front Public Health. 2022;10:1034227.

208. Ren J, Pang W, Luo Y, Cheng D, Qiu K, Rao Y, et al. Impact of Allergic Rhinitis and Asthma on COVID-19 Infection, Hospitalization, and Mortality. J Allergy Clin Immunol Pract. 2022;10(1):124-33.

209. Safizadeh F, Nguyen TNM, Brenner H, Schottker B. Association of renin-angiotensin-aldosterone system inhibition with Covid-19 hospitalization and all-cause mortality in the UK biobank. Br J Clin Pharmacol. 2022;88(6):2830-42.

210. Scalsky RJ, Chen YJ, Ying Z, Perry JA, Hong CC. The Social and Natural Environment's Impact on SARS-CoV-2 Infections in the UK Biobank. Int J Environ Res Public Health. 2022;19(1):533.

211. Schneider CV, Schneider KM, Teumer A, Rudolph KL, Hartmann D, Rader DJ, et al. Association of Telomere Length With Risk of Disease and Mortality. JAMA Intern Med. 2022;182(3):291-300.

212. Shawer H, Cheng CW, Bailey MA. Absence of association between host genetic mutations in the ORAI1 gene and COVID-19 fatality. PLoS One. 2022;17(2):e0263303.

213. Shcherbak SG, Changalidi AI, Barbitoff YA, Anisenkova AY, Mosenko SV, Asaulenko ZP, et al. Identification of Genetic Risk Factors of Severe COVID-19 Using Extensive Phenotypic Data: A Proof-of-Concept Study in a Cohort of Russian Patients. Genes (Basel). 2022;13(3):534.

214. Sheridan C, Klompmaker J, Cummins S, James P, Fecht D, Roscoe C. Associations of air pollution with COVID-19 positivity, hospitalisations, and mortality: Observational evidence from UK Biobank. Environ Pollut. 2022;308:119686.

215. Shi H, Zhao H, Zhang W, Wang S. COVID-19 is not a causal risk for miscarriage: evidence from a Mendelian randomization study. J Assist Reprod Genet. 2022:1-9.

216. Sims JM, Lawrence E, Glazer K, Gander A, Fuller B, Davidson BR, et al. Lessons learned from the COVID-19 pandemic about sample access for research in the UK. BMJ Open. 2022;12(4):e047309.

217. Sun Y, Chatterjee R, Ronanki A, Ye K. Circulating Polyunsaturated Fatty Acids and COVID-19: A Prospective Cohort Study and Mendelian Randomization Analysis. Front Med (Lausanne). 2022;9:923746.

218. Tan JS, Liu N, Guo TT, Hu S, Hua L, Qian Q. Genetic Predispositions Between COVID-19 and Three Cardio-Cerebrovascular Diseases. Front Genet. 2022;13:743905.

219. Thibord F, Chan MV, Chen MH, Johnson AD. A year of COVID-19 GWAS results from the GRASP portal reveals potential genetic risk factors. HGG Adv. 2022;3(2):100095.

220. Thomson A, Liu J, Bonnechère B, Amin N, Van Duijn CM. Disentangling the relationship between apolipoprotein E, cardiovascular disease, COVID-19, dementia and mortality. Alzheimer's & Dementia. 2022;17(S3):e056036.

221. Topless R, Green R, Morgan S, Robinson P, Merriman T, Gaffo A. Pos1222 Folic Acid and Methotrexate Use and Their Association with Covid-19 Diagnosis and Mortality: An Analysis from the Uk Biobank. Annals of the Rheumatic Diseases. 2022;81(Suppl 1):942.1-.

222. Topless R, Green R, Morgan SL, Robinson P, Merriman T, Gaffo AL. Folic acid and methotrexate use and their association with COVID-19 diagnosis and mortality: a case-control analysis from the UK Biobank. BMJ Open. 2022;12(8):e062945.

223. Topless RK, Gaffo A, Stamp LK, Robinson PC, Dalbeth N, Merriman TR. Gout and the risk of COVID-19 diagnosis and death in the UK Biobank: a population-based study. Lancet Rheumatol. 2022;4(4):e274-e81.

224. Varela DC, Sarmiento Monroy JC, Mantilla Ribero MJ, Villota C, Rodriguez-Jimenez M, Mantilla Hernández RD. Ab0314 Multimorbidity Burden in Rheumatoid Arthritis. Annals of the Rheumatic Diseases. 2022;81(Suppl 1):1282.1-.

225. Vu TT, Van Horn L, Achenbach CJ, Rydland KJ, Cornelis MC. Diet and Respiratory Infections: Specific or Generalized Associations? Nutrients. 2022;14(6):1195.

226. Wan TK, Huang RX, Tulu TW, Liu JD, Vodencarevic A, Wong CW, et al. Identifying Predictors of COVID-19 Mortality Using Machine Learning. Life (Basel). 2022;12(4):547.

227. Wilkinson TJ, Yates T, Baker LA, Zaccardi F, Smith AC. Sarcopenic obesity and the risk of hospitalization or death from coronavirus disease 2019: findings from UK Biobank. JCSM Rapid Commun. 2022;5(1):3-9.

228. Willette AA, Willette SA, Wang Q, Pappas C, Klinedinst BS, Le S, et al. Using machine learning to predict COVID-19 infection and severity risk among 4510 aged adults: a UK Biobank cohort study. Sci Rep. 2022;12(1):7736.

229. Xie J, Feng S, Li X, Gea-Mallorqui E, Prats-Uribe A, Prieto-Alhambra D. Comparative effectiveness of the BNT162b2 and ChAdOx1 vaccines against Covid-19 in people over 50. Nat Commun. 2022;13(1):1519.

230. Xie J, Prats-Uribe A, Feng Q, Wang Y, Gill D, Paredes R, et al. Clinical and Genetic Risk Factors for Acute Incident Venous Thromboembolism in Ambulatory Patients With COVID-19. JAMA Intern Med. 2022;182(10):1063-70.

231. Xie J, Prats-Uribe A, Gordillo-Maranon M, Strauss VY, Gill D, Prieto-Alhambra D. Genetic risk and incident venous thromboembolism in middle-aged and older adults following COVID-19 vaccination. J Thromb Haemost. 2022;20(12):2887-95.

232. Yanik EL, Evanoff BA, Dale AM, Ma Y, Walker-Bone KE. Occupational characteristics associated with SARS-CoV-2 infection in the UK Biobank during August-November 2020: a cohort study. BMC Public Health. 2022;22(1):1884.

233. Yoshiji S, Tanaka D, Minamino H, Lu T, Butler-Laporte G, Murakami T, et al. Causal associations between body fat accumulation and COVID-19 severity: A Mendelian randomization study. Front Endocrinol (Lausanne). 2022;13:899625.

234. Yoshiji S, Tanaka D, Minamino H, Lu T, Murakami T, Fujita Y, et al. LBODP001 Causal Associations Between Body Fat Accumulation and Covid-19 Severity: A Mendelian Randomization Study. Journal of the Endocrine Society. 2022;6(Supplement_1):A1-A.

235. Zhang H, Zhou Z. COVID-19 and the risk of Alzheimer's disease, amyotrophic lateral sclerosis, and multiple sclerosis. Ann Clin Transl Neurol. 2022;9(12):1953-61.

236. Zhou L, Li H, Zhang S, Yang H, Ma Y, Wang Y. Impact of ultra-processed food intake on the risk of COVID-19: a prospective cohort study. Eur J Nutr. 2022:1-13.

Figure S1: UK Biobank SARS-CoV-2 Serology Study

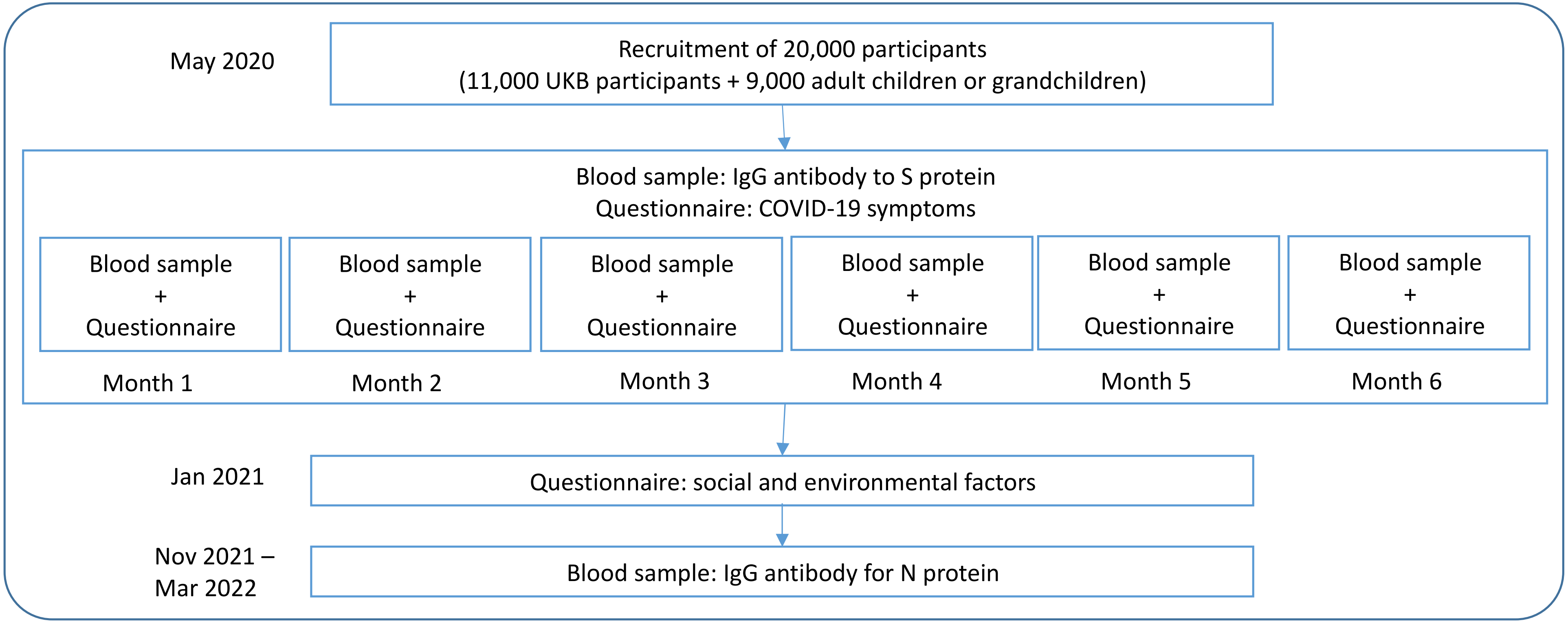

Figure S2: UK Biobank Coronavirus Self-Test Antibody Study

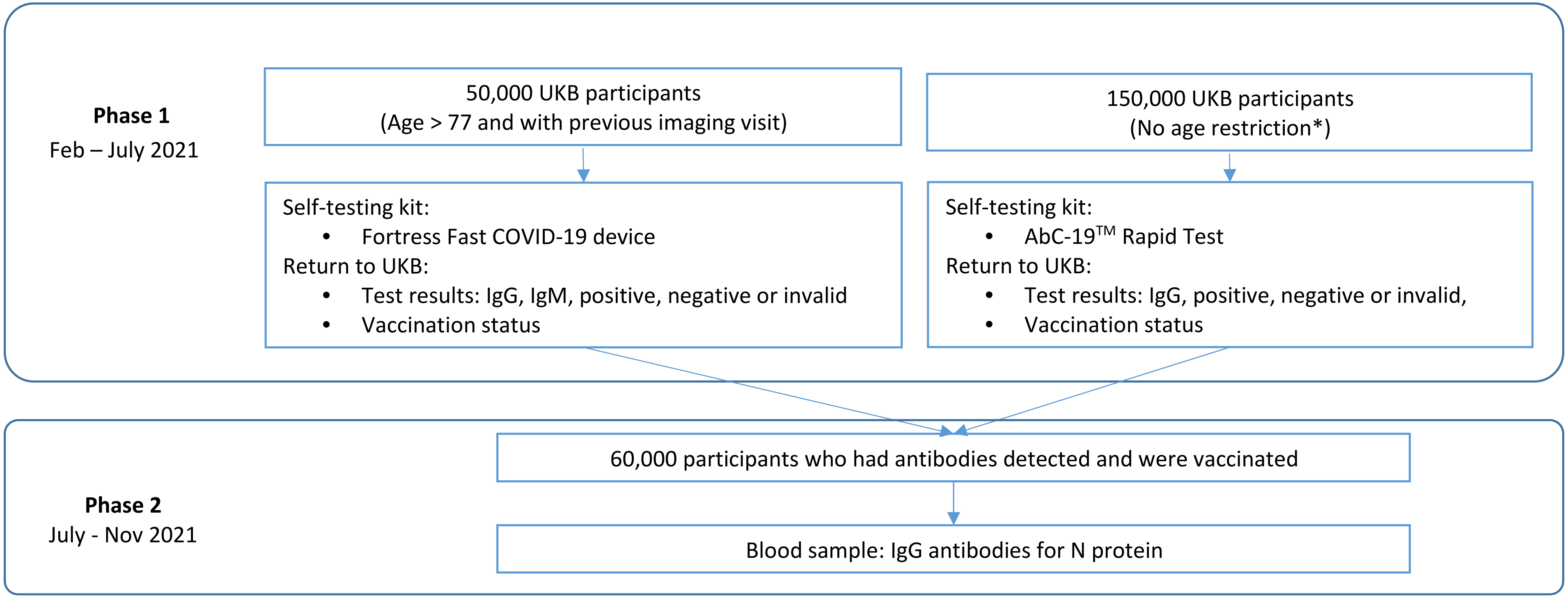

*: the inclusion criteria here: (1) participants have not taken part in the 50,000-participant phase 1 study, (2) participants were willing to be contacted by UK Biobank.
